# *Notice to Readers:* Special Podcast: “Defining Moments in *MMWR* History — *E. coli* O157:H7”

**DOI:** 10.15585/mmwr.mm6621a5

**Published:** 2017-06-02

*MMWR* has released a special podcast that highlights the leading role that *MMWR* played in reporting on the deadly multistate *Escherichia coli* O157:H7 foodborne outbreak of 1993. “Defining Moments in *MMWR* History – *E. coli* O157:H7” features an interview with Dr. Beth Bell conducted by *MMWR* Editor-in-Chief Dr. Sonja Rasmussen. Dr. Bell, who served as director of the National Center for Emerging and Zoonotic Infectious Diseases (NCEZID) from 2010 to 2017 and as an Epidemic Intelligence Service Officer during 1992–1994, was one of the first public health responders on the scene for this landmark public health emergency.

During the outbreak, four children died, and approximately 700 persons in four states became ill with severe and often bloody diarrhea. The first reports of CDC’s investigation into this outbreak were published in *MMWR* (*1*,*2*).

Additional information regarding the outbreak is available at https://www.cdc.gov/od/science/wewerethere. The podcast is available at https://www.cdc.gov/mmwr/mmwrpodcasts.html.
